# Context and semantic object properties interact to support recognition memory

**DOI:** 10.1177/17470218241283028

**Published:** 2024-09-28

**Authors:** Shirley Pandya, Victoria I Nicholls, Alexandra Krugliak, Simon W Davis, Alex Clarke

**Affiliations:** 1 Department of Psychology, University of Cambridge, Cambridge, UK; 2 Department of Psychology, University of Miami, Coral Gables, FL, USA; 3 Department of Neurology, Duke University, Durham, NC, USA; 4 Department of Psychology, University of Warwick, UK

**Keywords:** Congruency, conceptual statistics, objects, schema, scenes

## Abstract

We have a great capacity to remember a large number of items, yet memory is selective. While multiple factors dictate why we remember some things and not others, it is increasingly acknowledged that some objects are more memorable than others. Recent studies show semantically distinctive objects are better remembered, as are objects located in expected scene contexts. However, we know little about how object semantics and context interact to facilitate memory. Here we test the intriguing hypothesis that these factors have complementary benefits for memory. Participants rated the congruency of object-scene pairs, followed by a surprise memory test. We show that object memory is best predicted by semantic familiarity when an object-scene pairing was congruent, but when object-scene pairings were incongruent, semantic statistics have an especially prominent impact. This demonstrates both the item and its schematic relationship to the environment interact to shape what we will and will not remember.

## Introduction

Successful memory formation requires that we encode visual and semantic details about the environments we inhabit and consolidate episodic events composed of these scenes and their constituent objects. However, memory encoding is a selective process, and while we have a great capacity to remember a large number of items (e.g., Brady et al., 2008; Draschkow et al., 2019; Standing et al., 1970), memory encoding operations clearly favour certain relationships between items and their environment. Such schematic relationships lend context to the identity for individual items that promotes memory, and yet our understanding of the relevance of the interaction of context and what the item *is* to successful memory is still in its infancy. On one hand, a growing body of work has found that the same images tend to be remembered across individuals (Bainbridge, 2019), and that specific item properties can predict why some images tend to be more memorable than others (Bylinskii et al., 2015; Khosla et al., 2015). On the other hand, the schema congruency between an item and a context has a profound influence on item and source memory (Staresina et al., 2009). Thus, when considering memory for a particular object in an environment, two specific aspects might have a dominant impact on memory for that object—the intrinsic semantic properties of the object (e.g., distinctiveness, familiarity), and the relationship between the object and the surrounding scene (i.e., its schematic relationship). While the semantic properties and schema congruency of the object have both independently been used to predict subsequent memory, the interaction of context and semantics has not been formally assessed. Here we investigate the possibility that these two modulations of object memory confer complementary benefits.

Schema-based knowledge helps us understand how objects and scenes are interconnected based on how frequently they are paired together in the environment and enables us to predict what kinds of objects are likely in different settings. For instance, seeing a pan in a kitchen would be consistent with that schema, while it would be inconsistent, but not impossible, in a location like a garden. The spatiotemporal context in which we learn information is widely known to impact later memory for both scene and object-in-scene information. While memory for objects is better when objects are presented in isolation, rather than as part of a scene (Evans & Wolfe, 2022), the relationship between the object and its location also impacts memory. Typically, memory for objects is improved when they are studied in congruent rather than incongruent scenes (Brewer & Treyens, 1981; Frank et al., 2018; van Kesteren et al., 2013; but also see Greve et al., 2019). This congruency benefit is believed to be supported by schemas or situation models, built up over time as objects become associated with common contexts and meanings (Bartlett, 1932; van Kesteren et al., 2012; Võ, 2021). What is lost in these paradigms, and the majority of episodic memory studies, is that not all objects are equally memorable. Various item-wise properties including conceptual statistics promote memory, motivating a careful consideration of the relationship between the scene and the objects in addition to the meaning of the object.

An objects’ conceptual properties can include information about what broad kind of object it is (e.g., an animal, tool etc.), while conceptual measures can also vary on an item-wise level to capture the internal structure of a concept—that being, the properties a concept is composed of, and how those properties co-occur with one another across concepts. To help define the conceptual structure of object concepts, a number of studies have used distributed models of conceptual knowledge. Feature-based models define a concept based on property norming data (e.g., a tiger has the features “*has stripes*,” “*is found in India*”), from which various statistics can be calculated by taking advantage of the co-occurrence of features across concepts. For example, Naspi et al. (2021) calculated a measure of *conceptual confusability*, whereby concepts with more shared features with other concepts showed poorer mnemonic recognition performance (and conversely, concepts with more distinctive/unique features showed better memory). Recently, Hovhannisyan et al. (2021) found that images of concepts whose more distinctive features tended to be more strongly correlated with the other features of the concept (*correlation x distinctiveness*; CxD) showed better memory in both visual and lexical recognition memory tasks. Together, these studies argue that both the distinctiveness and the correlated nature of the conceptual properties influence memory. This highlights an important implication for future memory research—that an items conceptual structure will influence whether that item is better remembered or not and that memorability is not defined solely by the image itself, but by what that image *means*. The findings emerging from these studies suggest that distributed models of conceptual knowledge not only relate to conceptual processes as meaning is accessed (Clarke et al., 2013; Devereux et al., 2016; Taylor et al., 2012; Tyler et al., 2013), but extend to processes important for later memory (Davis et al., 2021; Hovhannisyan et al., 2021; Naspi et al., 2021).

Both the congruency effect and effects of conceptual structure statistics show that meaning has an influence on later memory. While we predict better recognition memory for objects in congruent than incongruent scenes, and better memory for objects with more distinctive conceptual structures, it remains to be seen how these two aspects interact to support recognition memory. In this study, we examined several questions regarding how context and semantics might influence object memory. Participants first studied various object-scene pairs and made congruency judgements for each pair, before performing a surprise recognition memory test for the objects and the scene the object was paired with. Based on the participant’s ratings for congruency, and conceptual structure statistics from a distributed model of semantics (Devereux et al., 2014; Moss et al., 2007; Taylor et al., 2011), we then sought to determine how these different factors contribute and interact to support later memory, and if this varied according to the format of memory retrieval (visual vs lexical recognition memory). The two types of memory retrieval task were chosen following Hovhannisyan et al. (2021) to explore how visual and lexical memory for items might be differentially impacted by contextual and conceptual factors, with the lexical task seen as a more direct measure of conceptual memory which cannot be driven by the presence of visual features during the test phase.

## Methods

### Participants

One hundred and two participants were recruited for the study (age range 18–36, mean 27 years, 23 male, 79 female) using the online platform Prolific (www.prolific.co). All participants were native English speakers based in the United Kingdom and had normal or corrected to normal vision. All participants provided informed consent to take part, and the study was approved by the Psychology Research Ethics Committee at the University of Cambridge. Participants were compensated £8 per hour for their time, and data were collected in July and August 2022.

### Stimuli and procedure

The study used a total of 398 unique images across the two phases of the study (139 objects, 150 scenes, 109 lure objects). The 139 images each showed a single object in colour at the centre of a white background. The 150 scenes were presented in colour, and depicted one of three categories—indoor domestic, outdoor urban, or outdoor rural. The scenes images were obtained from Lauer et al. (2018), the SUN397 scene image database (Xiao et al., 2010), and internet searches using Google Image Search. The 109 lure images showed a single object in colour at the centre of a white background and were taken from the DinoLab property norms set (Hovhannisyan et al., 2021).

**Figure 1.**
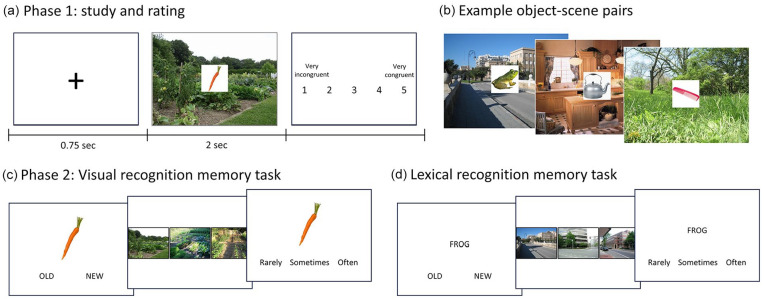
Study design. (a) During phase 1, participants see object-scene pairs for 2 s and then rate the congruency between the object and the scene. (b) Example object-scene pairs with varying degrees of expected congruency. In phase 2, participants either do a visual recognition memory task (c) or a lexical recognition memory task (d). In both memory tasks, participants are presented with an item and must choose whether it was old (i.e., seen in phase 1) or new. For all old responses, a choice of three similar scenes is presented and they must select which scene the item was paired with in phase 1. They then rate how often they see or used the item.

*Phase 1: Study and congruency ratings.* In phase 1, participants saw images of scenes with an object positioned centrally on top, and provided a congruency rating for the object-scene pairing (Figure 1a). Each participant viewed and rated the congruency of 109 object-scene pairs, where approximately half were more congruent and half were more incongruent. The object-scene pairings were created during a pretest with a separate group of 37 participants. The object-scene pairings were initially constructed from a larger set of 150 scenes and objects by the experimenters, so that each scene had an object that was more, and an object that was less consistent with it (and each object was paired with two scenes). In the pretest, participants rated each object-scene pair based on how likely they were to encounter this object in the scene, on a scale from 1 to 5 (1 = *very unlikely*, 2 = *unlikely*, 3 = *neutral*, 4 = *likely*, 5 = *very likely*). Object-scene pairs that did not receive a clear congruent or incongruent rating at the group level were reshuffled until 150 congruent and 150 incongruent object-scene pairings remained. Congruent pairs were defined by group average ratings greater than 3.5, and incongruent pairs were defined by average ratings of less than 1.5.

From this list of 300 object-scene pairs (where every object and scene appears twice), two shorter lists were created where neither the object nor the scene could be repeated, and where approximately half the pairs had an average congruent relationship and half had an average incongruent relationship (Figure 1b). When creating the lists, we attempted to maximise the number of objects and scenes that were present in both lists, meaning that many of the objects and scenes would be viewed in both a congruent and an incongruent pairing across participants (although each participant will only see each object and scene a maximum of once in phase 1). This resulted in two lists of 109 object-scene pairs, where 79 of the possible 150 objects appeared in both lists, and 68 of the possible 150 scenes appeared in both lists. Half of the participants saw object-scene pairings from list 1 and half of the participants saw pairings from list 2. Object-scene pairings can be seen at https://osf.io/rw92p/.

During phase 1, participants were asked to observe each image and indicate how congruent the object was with the scene. Each trial began with a fixation cross for 750 ms, before an object was shown with a scene for 2 s. Following this, the participant indicated the level of congruency of the object-scene pair using a 5-point Likert-type scale (1 = *very incongruent*, 2 = *incongruent*, 3 = *neutral*, 4 = *congruent*, 5 = *very congruent*). Participants completed all 109 trials before moving on to Phase 2, and all trials were presented in a randomised order.

*Phase 2: Recognition memory test.* The second phase of the study assessed item recognition memory, object-scene memory (source memory), and collected an additional rating of familiarity/object use (Figure 1c and d). Item recognition memory was assessed through an old/new memory judgement, while object-scene association memory was assessed through a 3AFC test where participants selected the scene image they believed was associated with the item. Both tests were self-paced. Item memory was assessed using all 109 objects from Phase 1, plus an additional 109 lure images taken from the DinoLab property norms (Hovhannisyan et al., 2021). The 3AFC source memory test used the same 109 scene images from Phase 1.

Each of the 218 trials began with a fixation cross for 750 ms, after which an object was shown with the options: ‘old’—meaning they had seen the object during study phase 1, or ‘new’—if they had not seen the object before. For trials where participants chose “old,” the next screen displayed three different scene images and participants had to select the scene they thought the object was paired with in Phase 1. For cases where the item was truly old, one of the images would be correct, and the other two images were exemplars from the same scene category. If the participant responded “old” but the image was new, then three images from a single scene category were randomly selected. Finally, for all trials, regardless of item memory choice, participants ranked how often they see or use the object in their daily lives, on a 3-point Likert-type scale (i.e., familiarity). Trials were presented in a randomised order for every participant.

In Phase 2, we additionally manipulated the format the object was shown in. Half the participants (*N* = 51) were shown the same visual image of object used in Phase 1, while the remaining half (*N* = 51) were shown a written word on the screen denoting the concept name, and did not see a visual depiction of the object. This allowed us to examine visual recognition memory in half the participants (Figure 1c), and lexical recognition memory (Figure 1d) in the remaining half. Both phases of the study were designed and run using Gorilla (www.gorilla.sc), with participants restricted to using a desktop computer and a time limit of 60 min. Median time to complete the study was 30 min. While all 102 participants completed the object recognition memory test, the scene recognition test reported was only administered to half the participants (*N* = 50).

### Statistical modelling

Our primary analyses used generalised logistic linear mixed-effects models (fitglme) implemented in Matlab R2020b. Recognition accuracy for the old items in phase II were modelled using a Binomial distribution. The potential fixed-effects included, whether the participant completed a visual or lexical recognition memory task in phase II; the congruency score between the scene and the object, as indicated by the participant in phase I on a 5-point scale; the familiarity the participant had with the item, rated on a 3-point scale; and three semantic measures for each object: mean distinctiveness (MD), correlational strength (CS), and CxD. All fixed effects were treated as continuous measures, and all models included random effects of subject, object, and scene image.

The three semantic measures were chosen due to their prior association with conceptual processing across visual and language stimuli (Clarke et al., 2013; Devereux et al., 2016; Taylor et al., 2012; Tyler et al., 2013) and their influence on recognition memory (Hovhannisyan et al., 2021). All measures were calculated from the CSLB property norms (Devereux et al., 2014) which contain 826 concepts and 3026 features. MD is a concept-level statistic, which is the mean across the feature distinctiveness values for the features associated with that concept. For each of the 3026 features in the property norms, we calculated feature distinctiveness as 1/Nr concepts associated with that feature, where higher numbers indicate that the feature is relatively distinctive for a concept, and high MD values indicates that the concept has relatively distinctive features associated with it. CS reflects the tendency for features associated with a concept to co-occur with one another across different concepts. For example, the features <has fur>, <has eyes> and <has a tail> tend to co-occur across many animals. First the correlation between all pairs of feature vectors in a concept is calculated and significantly correlated (*p* < .05) feature–feature vectors are retained. CS is then the mean correlation between features of that concept, with high scores indicating that the concepts’ features tend to co-occur with one another across concepts. CxD is a measure that captures whether a concept’s more distinctive features also tend to be correlated within the concept. For each concept, CxD is calculated from a scatterplot of the concept’s features, with feature distinctiveness on the y-axis and mean CS for the feature across all concepts on the x-axis. The CxD value is the unstandardized beta coefficient for mean CS regressed against distinctiveness. A higher value indicates that a concept’s more distinctive features are more highly correlated, and lower values suggest a concept’s more shared features are more highly correlated. For all measures, features occurring in two or fewer concepts are excluded.

The three semantic variables were normalised using the Yeo Johnson transformation and converted to z-scores. Normality was assessed using the Shapiro–Wilk’s test. Normalisation and normality test were conducted using JASP (version 0.16.4). Based on the semantic measures, items with z-scores beyond ±3 were excluded from the models to avoid the influence of outliers. Pearson’s correlation was calculated between all potential predictors in the mixed-effects models (Table 1), showing little relationship between congruency judgements and the other measures. To formally asses the collinearity of the predictors, variance inflation factors were calculated, with all variables having a value less than 2 (values over 5 suggest the model is biased due to multicollinearity). Plots of the fixed effects and interactions used the residualised scores from different mixed-effects models to illustrate the unique relationships between predictors and outcome measures.

**Table 1.** Correlations between model predictors.

	Congruency	Familiarity	MD	CS	CxD
Congruency	-	0.14	−0.05	−0.03	−0.02
Familiarity		-	−0.04	−0.02	−0.18
Mean distinctiveness (MD)			-	0.61	0.20
Correlational strength (CS)				-	0.20
Correlation x distinctiveness (CxD)					-

### Transparency and openness

The study reported here was not pre-registered. All stimuli, data, and code associated with this research can be freely accessed at https://osf.io/rw92p/.

## Results

### Overall memory performance

We first assessed overall memory performance, as measured by the participants’ ability to (1) distinguish between old and new objects, (2) whether they associated the objects with the correct scene image (source memory), and (3) whether performance varied across the visual and lexical recognition tasks (Table 2). Object recognition performance across hit rates, false alarms, and d prime all suggest that participants were able to successfully discriminate between “old” and “new” objects in both tasks, with no significant task differences seen in d prime, *t*(100) = 1.40, *p* = .164, hit rate, *t*(100) = 1.00, *p* = .321, or false alarms, *t*(100) = 0.35, *p* = .731. Critically, hit rates showed a large proportion of the objects were judged as *new* when they were in fact *old*, which is important to enable us to test what factors lead to successful and unsuccessful recognition memory at the item-level.

**Table 2.** Mean behavioural performance on the visual and lexical recognition tasks.

	Hit rate	False alarms	d’	Item only	Item + Scene	Item misses
Visual task	0.71 (0.124)	0.06 (0.086)	2.39 (0.602)	0.20 (0.069)	0.51 (0.148)	0.29 (0.135)
Lexical task	0.69 (0.133)	0.06 (0.066)	2.22 (0.650)	0.21 (0.063)	0.49 (0.111)	0.30 (0.103)

Hit rate, false alarms and d’ based on item recognition memory test. Item only rate is proportion of all “old” trials where the item was correctly identified as “old” but with an incorrect scene judgement. Item + Scene rate is proportion of all “old” trials where both the item and scene were correct. Item misses is the proportion of all “old” trials responded to with a “new” judgement. Standard deviations shown in parentheses.

We next examined memory performance for the old items according to whether they were correctly identified along with the scene that the object was paired. Like for item recognition memory, no significant task differences were seen for item-only rate, *t*(48) = 0.36, *p* = .72, item + scene rate, *t*(48) = 0.33, *p* = .74, or item misses, *t*(48) = 0.16, *p* = .87.

### Item recognition

#### Influence of object-scene congruency on visual and lexical recognition

**Figure 2.**
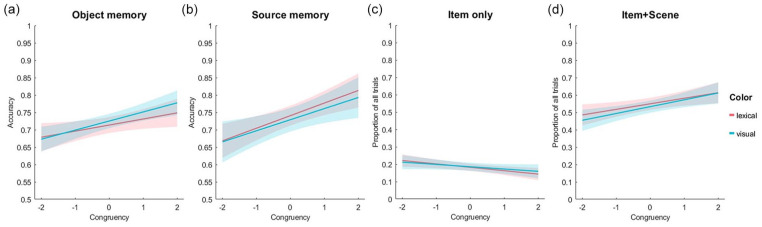
Recognition memory accuracy rates for different levels of object-scene congruency: (a) overall accuracy for object recognition memory for the visual and lexical recognition memory test. (b) source memory accuracy from the 3AFC scene memory test following successful object memory. (c) Proportion of all trials (item-only, item + scene, item misses) where the object was correctly remembered as old, but scene memory was inaccurate. (d) Proportion of all trials where both the object and scene memory was accurate. Lines show a linear fit to the data, with shaded areas showing 95% confidence intervals.

We next tested if recognition accuracy for “old” objects related to the congruency between the object and the scene the object was seen with in phase 1, and whether this relationship varied by task. A logistic linear mixed-effects model, with fixed effects of congruency, task (visual or lexical) and their interaction, showed no significant effects of task (beta estimate = −0.10, *t* = −0.79, *p* = .429) or any interaction between task and congruency (beta estimate < .01, *t* = .10, *p* = .921). As we found no evidence that the task influenced recognition memory, we ran a further linear mixed-effects model which only included the fixed effect of congruency, and used all data across both tasks (note that task was a between subjects factor). This revealed a significant positive effect of congruency on accuracy (beta estimate = .132, *t* = 10.21, *p* < .0001) with an approximate 10% increase in memory for objects in highly congruent compared to very incongruent contexts (Figure 2a). As follow-up analyses, this relationship was assessed for the visual and lexical tasks separately, showing similar significant effects of congruency in both tasks (Visual task: beta estimate = 0.130, *t* = 7.05, *p* < .0001; lexical task: beta estimate = 1.33, *t* = 7.37, *p* < .0001). This relationship replicates prior findings where higher object-scene congruency relates to better recognition memory for the objects (van Kesteren et al., 2013).

#### Item memory is influenced by congruency, familiarity, and the semantic properties of the item

After observing positive effects of congruency on item memory for both tasks (visual and lexical), we tested for any effects of the objects’ semantic properties on accuracy, in addition to congruency and familiarity (how often do you come across this object). In a further test of task differences and interactions, an initial linear mixed-effects model with fixed effects of task (visual or lexical), congruency, familiarity (rated from 0 to 2), and the semantic measures—MD, CS, CxD, was performed. Like before, there were no significant effects of task (beta estimate = −0.102, *t* = −0.709, *p* = .478) or interactions involving task (all *p* values > 0.1), and so this fixed effect was removed.

**Figure 3.**
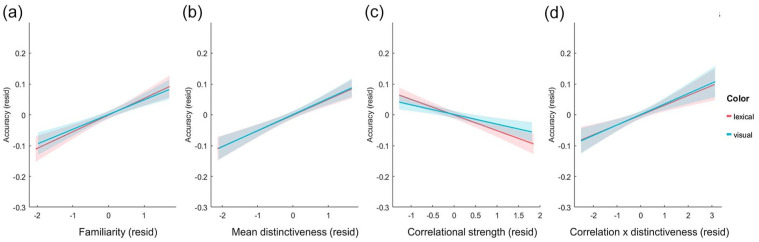
Residualised plots of object accuracy against the different predictors: (a) Familiarity, (b) Mean distinctiveness, (c) Correlational strength, and (d) Correlation x Distinctiveness. All measure on the x-axis are residualised against the other measures to show unique effects. Linear regression fitted lines displayed for visual and lexical tasks separately along with 95% confidence intervals.

A second linear mixed-effects model included fixed effects of congruency, familiarity, MD, CS, and CxD, in addition to interactions between congruency and the other predictors (Table 3). There remained a significant positive effect of congruency (beta estimate = 0.05, *t* = 2.25, *p* = .0247), and an additional significant positive effect of familiarity (beta estimate = .24, *t* = 7.35, *p* < .0001; Figure 3a). As for the semantic properties, we observed significant positive relationships between item accuracy and MD (beta estimate = 0.24, *t* = 7.79, *p* < .0001; Figure 3b) and CxD (beta estimate = 0.18, *t* = 6.13, *p* < .0001; Figure 3d), and a negative relationship between accuracy and CS (beta estimate = −0.19, *t* = −6.02, *p* < .0001; Figure 3c). Taken together, the main fixed-effects suggest that item memory is enhanced when the object was more congruent with the scene and the participant is more familiar with the object concept. Equally, recognition memory is higher when an object concept has more distinctive properties (positive effect of MD), those properties are less correlated within the concept (negative effect of CS), and when the more distinctive properties are more highlight correlated (positive effect of CxD). This kind of semantic statistical structure—where a concept’s properties are more distinctive, less correlated overall, but whose more distinctive features are relatively more correlated than the shared features, is more common for objects that are tools or vehicles and less common for living things.

**Table 3.** Fixed-effects results for the linear mixed-effects model of object recognition memory.

Predictor	Estimate	*SE*	T-value	*p* value	lower CI	Upper CI
Congruency	0.05	0.023	2.25	0.0247	0.01	0.10
Familiarity	0.24	0.032	7.35	<0.0001	0.17	0.30
Mean distinctiveness (MD)	0.24	0.031	7.79	<0.0001	0.18	0.30
Correlational strength (CS)	−0.19	0.032	-6.02	<0.0001	–0.25	–0.13
Correlation x distinctiveness (CxD)	0.18	0.030	6.13	<0.0001	0.13	0.24
Congruency: Familiarity	0.07	0.017	4.03	0.0001	0.04	0.10
Congruency: MD	0.01	0.018	0.33	0.7412	–0.03	0.04
Congruency: CS	0.05	0.019	2.71	0.0067	0.01	0.09
Congruency: CxD	−0.04	0.017	−2.20	0.0281	–0.07	–0.00

*Model df* *=* *9986.*

In addition to the main effects of each predictor, there were also significant interactions between congruency and familiarity (beta estimate = 0.07, *t* = 4.03, *p* = .0001), congruency and CS (beta estimate = .05, *t* = 2.71, *p* = .0067) and congruency and CxD (beta estimate = −0.04, *t* = −2.20, *p* = .0281). To understand these interactions, we plotted the data for each predictor at the different levels of congruency. To isolate the relationship of each predictor with recognition accuracy, we used linear mixed-effects models to extract the residual effects by controlling for all other variables except the measure being plotted. This shows how effects of familiarity, CS and CxD vary in impact across congruency rating, while controlling for the influence of the other variables.

**Figure 4.**
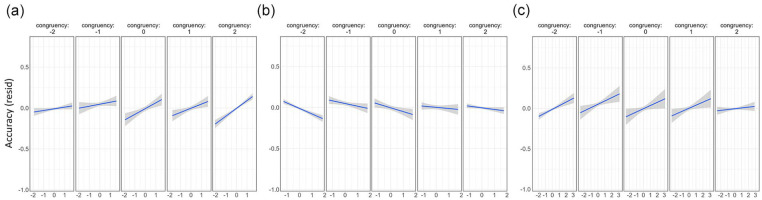
Residualised plots comparing the influence of familiarity and semantic properties as congruency differs. (a) Familiarity, (b) Correlational strength and (c) Correlation x Distinctiveness. All measures on the x-axis are residualised against the other measures to show the unique effects. Linear regression fitted lines along with 95% confidence intervals.

These plots show that the significant interaction between familiarity and congruency is driven by higher positive correlations with memory as the congruency rating increases (shown by steeper slopes for higher congruency levels), while familiarity has less influence on memory when objects were less congruent with the scene (Figure 4a). Conversely, CS and CxD appear to be more related to memory when the congruency between the object and the scene is lower (steeper slopes for lower congruency levels; Figure 4b and c). Going beyond the main effects of each measure, these results reveal several intriguing interactions between congruency, familiarity, and semantic properties in support of memory, with familiarity more important for memory when objects and scenes are congruent, and semantic properties more important when the object and scene is less congruent. Importantly, what an object is, and where it is seen, both interact to predict later memory.

As an additional control analysis, we re-ran the linear mixed-effects model including a regressor for naming agreement. This measure reflects how often the correct name was given for each object image by an independent group of participants. The measure was only available for 92% of our trials, and average name agreement was high (89.5%). The linear mixed-effects model showed no significant effects of name agreement (beta estimate < 0.001, *t* = 0.79, *p* = .429), and the pattern of effects across the other predictors mirrored those in Table 3.

### Source memory

#### Influence of object-scene congruency on source memory

In addition to object memory, we also tested source memory for the correctly remembered objects using a 3 AFC scene memory test. Following the same analyses as for object memory, we first tested for a relationship between source memory and object-scene congruency, and if this varied across the two tasks. Correct source memory was indicated by an accurate response for both the object and scene, and incorrect source memory was indicated by an incorrect response for the scene following a correct response for the object. A linear mixed-effects model with fixed effects of congruency and task revealed no significant effect of task (beta estimate = −0.09, *t* = −0.68, *p* = .498) or interaction of task and congruency (beta estimate = .036, *t* = .838, *p* = .402) on source memory. Note that we would not expect a task effect here given that all scenes were presented as images regardless of whether the object was shown as an image or a word. A second linear mixed-effects model with fixed effect of congruency revealed a significant positive effect of congruency on source accuracy (beta estimate = .156, *t* = 7.19, *p* < .0001) showing that memory for scenes was better for more congruent object-scene relationships (Figure 2b and d).

As an addition analysis, we further assessed the relationship between item-only trials and object-scene congruency (Figure 2c). A linear mixed-effects model with fixed effects of congruency and task revealed no significant effect of congruency (beta estimate = −0.045, *t* = 1.34, *p* = .179), task (beta estimate = −0.03, *t* = −0.19, *p* = .848) or interaction of task and congruency (beta estimate = −0.030, *t* = −0.62, *p* = .529) on item-only memory. The lack of a congruency effect on item-only trials is expected given that participants did not recognise the correct scene the object was paired with.

#### Source memory is influenced by congruency and semantic object properties of the item

**Figure 5.**
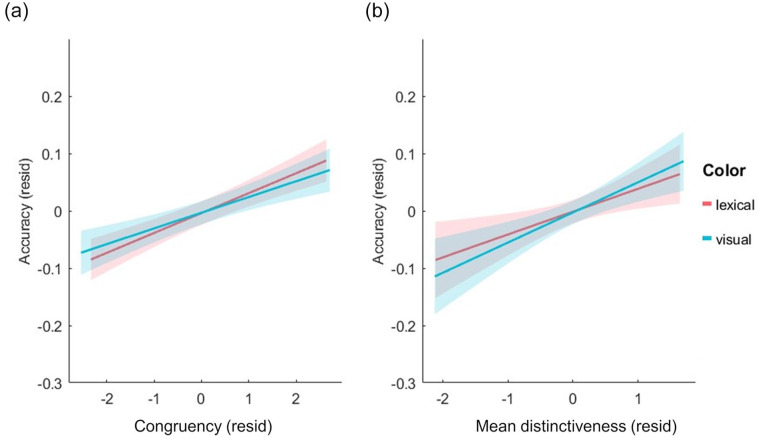
Residualised plots of source accuracy against the different predictors. (a) Congruency and (b) Mean distinctiveness. Measure on the x-axis are residualised against the other measures to show unique effects. Linear regression fitted lines displayed for visual and lexical tasks separately along with 95% confidence intervals.

We next examined the influence of congruency, and the semantic properties of the object on source memory (Table 4). This linear mixed-effects model showed a positive effect of congruency on source accuracy (beta estimate = 0.16, *t* = 6.86, *p* < .0001; Figure 5a), and a positive effect of MD of the object concept (beta estimate = 0.215, *t* = 3.98, *p* = .0001; Figure 5b). We observed no other significant effects or interactions. Both effects were also present for object recognition memory, meaning that memory for the object-scene pairing as a whole was best when the object and scene were congruent with one another and that memory for both the object and the scene were better when the object had more distinctive semantic properties.

**Table 4.** Fixed-effects results for the linear mixed-effects model of source memory.

Predictor	Estimate	*SE*	T value	*p* value	Lower CI	Upper CI
Congruency	0.16	0.024	6.86	<0.0001	0.12	0.21
MD	0.22	0.054	3.98	0.0001	0.11	0.32
CS	−0.07	0.055	−1.33	0.1829	−0.18	0.03
CxD	0.10	0.050	1.89	0.0591	−0.00	0.19
Congruency: MD	0.06	0.031	1.79	0.0733	−0.01	0.12
Congruency: CS	0.00	0.033	0.12	0.9035	−0.06	0.07
Congruency: CxD	0.01	0.028	0.25	0.8030	−0.05	0.06

DoF = 3425.

## Discussion

In this research, we explored a novel factor that supports object and scene memory, namely the interaction between object-scene congruency and the multifaceted conceptual properties of objects. In the first part of the study, participants studied and rated the congruency of object-scene pairs before a surprise recognition memory test for both the object and the scene. Furthermore, half the participants were engaged in a visual recognition memory test, and the remaining half a lexical recognition memory test. While we found no evidence of task differences on memory, we discovered that greater object-scene congruency led to improved memory for the object and the scene in both lexical and visual recognition tasks. In addition to the positive effect of congruency, we found that object memory was enhanced by greater familiarity with the object, as well as modulations of memory according to conceptual structure. Broadly, these findings replicated previous research on object-scene congruency (van Kesteren et al., 2012) and better memory for more conceptually distinctive objects (Hovhannisyan et al., 2021; Naspi et al., 2021), but crucially we were able to examine the interactions between factors, showing that familiarity has a more prominent role in object memory when the object-scene pairing is more congruent, while conceptual structure is more prominent when the object-scene pairing is less congruent.

Our finding that congruent objects and scenes were both better remembered is in line with a large body of work on recognition memory for schema consistent item pairs (Delhaye et al., 2023; Höltje et al., 2019; Staresina et al., 2009; van Kesteren et al., 2013). This semantic congruency benefit is argued to reflect the integration of an item with a consistent schema through mutual co-activation of semantic relationships between the item and context (Staresina et al., 2009) which can also be multi-sensory in nature (Duarte et al., 2023). These integrative processes will then facilitate retrieval (Greve et al., 2019), and as a consequence, better memory is observed for both objects and scenes from more congruent trials, as found in our data. However, the literature on such schema-congruent effects typically fails to account for item-level properties that may fundamentally influence the memorability of each object or object concept (Hovhannisyan et al., 2021), leading us to further explore how the conceptual properties of objects influence memory.

### The role of conceptual distinctiveness

Our findings demonstrate that item memory can be reinforced by a combination of congruency with the scene, having a more distinctive conceptual structure, and by our individual familiarity with a concept. Our main effects point to item memory being better when the concepts’ features were more distinctive, the shared features were less correlated with each other within the concept, and the distinctive features were relatively more correlated within the concept. Various factors of stimulus distinctiveness, in one form or another, have previously been linked to better memory (Reed Hunt, 2006). Here, we found a main effect of MD, which is a measure derived from a property norming study using words (Devereux et al., 2014) and provides a quantifiable measure of conceptual distinctiveness for basic-level categories that is unconnected to the visual images or stimulus set used. These findings are broadly in line with prior studies, which used words, that find atypical concepts benefit from higher recognition memory accuracy (Alves & Raposa, 2015; Delhaye et al., 2023; Souza et al., 2022), where an atypical item is described as a concept with more distinctive and fewer shared features with other members of its superordinate category (e.g., a penguin is an atypical bird). While these different studies all suggest having a more distinctive conceptual structure facilitates memory, our use of distinctiveness offers a significant benefit: unlike typicality, MD does not rely on an explicit hierarchical category structure, as it is computed relative to the entire conceptual space. This is beneficial, as many items do not belong to a clear conceptual category (e.g., a light bulb or a book), making the utility of typicality uncertain beyond clearer hierarchical categories. In contrast, the conceptual structure statistics associated with distributed models of conceptual knowledge are applicable to any concept (Moss et al., 2007; Taylor et al., 2011).

Alongside MD, we also tested measures of CS and CxD. Alongside this research, two other recent studies have harnessed conceptual structure statistics to try and explain object memory. Naspi et al (2021) reported that concepts that are more highly conceptually confusable—that is, the tendency of a concept to activate other similar concepts via shared features—are associated with poorer recognition. Conversely, this means that concepts with more distinctive conceptual features showed better memory, as found in our analysis. Another recent study utilising conceptual structure statistics and showed that delayed recognition memory was better for concepts whose more distinctive features were more correlated (Hovhannisyan et al., 2021), again similar to our effects, this time for CxD. This points to better memory for concepts with multiple distinctive features that themselves are highly linked. Our study, along with these two examples, highlight how conceptual structure statistics can be used to not only explain the perception and processing of concepts (Clarke et al., 2013; Tyler et al., 2013), but they also extend to help explain later memory for concepts.

Why should a more distinctive conceptual structure lead to better recognition memory? Another way to characterise the direction of the benefit of these three conceptual statistics (*more* distinctive features, *less* correlated shared features, and *higher* CxD), is by characterising concepts in a multidimensional space. For example, if a concept’s position within a semantic space is defined by its semantic feature relationships to other concepts, then concepts with many shared and correlated features will occupy a dense area of semantic space. It may therefore be easier to remember a bird like a toucan better than a sparrow, because the former concept lies further outside a tight cluster of “bird” concepts due to its highly distinctive, large beak (a feature that is not widely shared). Intuitively, conceptual confusability (Naspi et al., 2021) reflects the semantic density of a high-dimensional space; however, feature overlap is not the only factor contributing to how semantic spaces could be defined. Our results suggest that it is not only the degree to which a concept shares features that defines the structured representational space but also the correlated structure across those features. Memory traces for items which have more shared conceptual features will become more similar to one another through semantisation of the trace, leading to poorer memory performance (Heinen et al., 2024), and increased false memories when items are more closely semantically related (Roediger et al., 2001). This may mean that the reason conceptually distinctive objects are better remembered is because this measure increases for items in less dense areas of semantic space—a space represented in the ventral visual and frontal cortices, and memory traces in less dense representational spaces benefit from reduced interference, in turn supporting the retrieval of that item. Some evidence for this role of shared features playing a role in memory success is suggested by fMRI studies employing both representational similarity and subsequent memory approaches (Davis et al., 2021), which have found that several ventral temporal and parietal regions associated with object representations show greater correlations between neural similarity and shared semantic features (e.g., similarity in conceptual feature overlap). In other words, the effective representation of a dense semantic space in these regions has positive outcomes for memory, supporting the notion that such semantic processing through representational geometries contributes to lasting memory traces. This may be further facilitated through the integration of perceptual and conceptual features in the anterior temporal lobe (Martin et al., 2018).

### Interactions between schematic and conceptual factors

Perhaps the most exciting finding in these data was evidenced by a significant interaction between congruency and conceptual stimulus properties. Both the CxD measure and CS measure significantly interacted with congruency, in that they had maximal effects when object-scene congruency was perceived as being low. Past studies testing recognition memory for typical and atypical lexical concepts following a congruent or incongruent category cue have reported mixed results, where independent effects of typicality and congruency are seen (Alves & Raposa, 2015; Delhaye et al., 2023), or no influence of typicality (Höltje et al., 2019). However, one study does suggest that recognition memory benefits for atypical items might be stronger in incongruent trials than congruent trials (Alves & Raposa, 2015). Our measure most closely related to typicality (MD) did not show such an interaction with congruency. In contrast, more comprehensive measures of feature organisation—CS and CxD—both showed positive effects on memory for more incongruent trials.

Objects found in unexpected scenes are associated with longer recognition times and reduced recognition accuracy (Davenport & Potter, 2004; Greene et al., 2015; Oliva & Torralba, 2007; Palmer tephen, 1975) along with extended neural semantic processing during perception (Krugliak et al., 2023). This enhanced semantic processing of objects in incongruent scenes may require conceptual structure to play a larger role in memory, as the lack of familiarity with this novel schema (or object-scene pairing) may fail to facilitate memory as it would do for schema-consistent trials. Conversely, how familiar the participant was with the concept had a maximal positive effect for schema-congruent trials. Overall, familiarity showed a positive relationship with recognition memory. This suggests that having greater interactions with a concept increases the ability to remember that item, similar to enhanced visual memory for items in experts (Evans et al., 2011). However, familiarity also interacted with congruency, where familiarity had maximal effects for the more congruent object-scene pairs. Concepts which are more familiar to an individual will form a stronger part of a semantic schema or situation model, and so when they are perceived in a scene consistent with that schema, there will be a stronger facilitation of the semantic congruency effect. Taken together, this pattern concerning familiarity and conceptual structure measures suggests a complimentary system by which items-in-context are best remembered: when an object-scene pairing is intuitive (congruent), memory can be best predicted by participants’ familiarity with that common schema. However, when object-scene pairings violate expectations (incongruent trials), the underlying conceptual structure of item concepts has an especially prominent impact on memory, given the surprising association between an object and its surrounding environment.

One influencing factor that we did not explore in this study was the influence of visual image statistics and features on later memory. Various studies have found image properties to influence object memorability (e.g., Bylinskii et al., 2015; Khosla et al., 2015), with Hovhannisyan et al. (2021) including both visual and conceptual measures in their analyses of recognition memory. In addition to effects relating to the same conceptual structure measure we report, they also found that visual statistics extracted from deep neural networks trained on object images predicted memory success for both visual and lexical recognition tasks, pointing to independent visual and conceptual effects on memory. In this study, we chosen not to include visual statistics as predictors to focus on the interaction of context and conceptual measures, and reduce the models complexity. However, targeted visual statistics could be assessed in future work to see what explanatory value they have above the measures included.

Finally, while our focus here was on object memory, and memory for the associated scene, future work could further assess scene recognition memory along with memory for the associated object(s). While scene memory is expected to be lower overall than object recognition memory (Evans & Wolfe, 2022), shifting the focus to the scene allows us to not only test the semantic relationships between scenes and objects, but additionally the location of the object within the scene. This would be facilitated through embedding objects into scenes, rather than having objects isolated, with the prediction that a U-shaped effect between expectedness of location and accuracy would emerge (Quent et al., 2022). How semantic measures relating to scene and object content interact with such an effect remains unexplored.

## Conclusion

This analysis demonstrates that the semantic relationships between items and their environments have a strong influence on later memory. While there is a powerful influence of whether the items we experience are consistent with our schematic knowledge of that environment, facilitated by our familiarity with that item, we also show that what the item *means* contributes to memory. We find that when the object and context fit with our schematic knowledge, then this is the prevailing influence on memory, while when objects do not fit into our existing schemas, then the conceptual distinctiveness becomes influential for memory. This suggests that schematic and conceptual knowledge about objects can have complimentary functions in facilitating later memory.

## References

[bibr1-17470218241283028] Alves M. Raposa A. (2015). Is it a bird? Differential effects of concept typicality on semantic memory and episodic recollection. Revista Portuguesa de Psicologia, 44, 65–79.

[bibr2-17470218241283028] Bainbridge W. A. (2019). Chapter one—memorability: How what we see influences what we remember. In Federmeier K. D. Beck D. M. (Eds.), Psychology of learning and motivation ( Vol.70, pp. 1–27). Academic Press. 10.1016/bs.plm.2019.02.001

[bibr3-17470218241283028] Bartlett F. C. (1932). Remembering: A study in experimental and social psychology (pp. xix, 317). Cambridge University Press.

[bibr4-17470218241283028] Brady T. F. Konkle T. Alvarez G. A. Oliva A. (2008). Visual long-term memory has a massive storage capacity for object details. Proceedings of the National Academy of Sciences of the United States of America, 105(38), Article 38. 10.1073/pnas.0803390105 PMC2533687 18787113

[bibr5-17470218241283028] Brewer W. F. Treyens J. C. (1981). Role of schemata in memory for places. Cognitive Psychology, 13(2), 207–230. 10.1016/0010-0285(81)90008-6

[bibr6-17470218241283028] Bylinskii Z. Isola P. Bainbridge C. Torralba A. Oliva A. (2015). Intrinsic and extrinsic effects on image memorability. Vision Research, 116, 165–178. 10.1016/j.visres.2015.03.005 25796976

[bibr7-17470218241283028] Clarke A. Taylor K. I. Devereux B. Randall B. Tyler L. K. (2013). From perception to conception: How meaningful objects are processed over time. Cerebral Cortex, 23(1), Article 1. 10.1093/cercor/bhs002 PMC3619663 22275484

[bibr8-17470218241283028] Davenport J. L. Potter M. C. (2004). Scene consistency in object and background perception. Psychological Science, 15(8), Article 8. 10.1111/j.0956-7976.2004.00719.x 15271002

[bibr9-17470218241283028] Davis S. W. Geib B. R. Wing E. A. Wang W.-C. Hovhannisyan M. Monge Z. A. Cabeza R. (2021). Visual and semantic representations predict subsequent memory in perceptual and conceptual memory tests. Cerebral Cortex, 31(2), Article 2. 10.1093/cercor/bhaa269 PMC8485078 32935833

[bibr10-17470218241283028] Delhaye E. Coco M. I. Bahri M. A. Raposo A. (2023). Typicality in the brain during semantic and episodic memory decisions. Neuropsychologia, 184, 108529. 10.1016/j.neuropsychologia.2023.108529 36898662

[bibr11-17470218241283028] Devereux B. J. Taylor K. I. Randall B. Geertzen J. Tyler L. K. (2016). Feature statistics modulate the activation of meaning during spoken word processing. Cognitive Science, 40(2), 325–350. 26043761 10.1111/cogs.12234 PMC4949631

[bibr12-17470218241283028] Devereux B. J. Tyler L. K. Geertzen J. Randall B. (2014). The Centre for Speech, Language and the Brain (CSLB) concept property norms. Behavior Research Methods, 46(4), Article 4. 10.3758/s13428-013-0420-4 PMC4237904 24356992

[bibr13-17470218241283028] Draschkow D. Reinecke S. Cunningham C. A. Võ M. L.-H. (2019). The lower bounds of massive memory: Investigating memory for object details after incidental encoding. Quarterly Journal of Experimental Psychology, 72(5), Article 5. 10.1177/1747021818783722 29862888

[bibr14-17470218241283028] Duarte S. E. Ghetti S. Geng J. J. (2023). Object memory is multisensory: Task-irrelevant sounds improve recollection. Psychon Bull Rev 30, 652–665. 10.3758/s13423-022-02182-1 36167915 PMC10040470

[bibr15-17470218241283028] Evans K. K. Cohen M. A. Tambouret R. Horowitz T. Kreindel E. Wolfe J. M. (2011). Does visual expertise improve visual recognition memory? Attention, Perception, & Psychophysics, 73(1), 30–35. 10.3758/s13414-010-0022-5 PMC3140200 21258906

[bibr16-17470218241283028] Evans K. K. Wolfe J. M. (2022). Sometimes it helps to be taken out of context: Memory for objects in scenes. Visual Cognition, 30(4), 229–244. 10.1080/13506285.2021.2023245

[bibr17-17470218241283028] Frank D. Montaldi D. Wittmann B. Talmi D. (2018). Beneficial and detrimental effects of schema incongruence on memory for contextual events. Learning & Memory, 25(8), 352–360. 10.1101/lm.047738.118 30012880 PMC6049394

[bibr18-17470218241283028] Greene M. R. Botros A. P. Beck D. M. Fei-Fei L. (2015). What you see is what you expect: Rapid scene understanding benefits from prior experience. Attention, Perception, & Psychophysics, 77(4), 1239–1251. 10.3758/s13414-015-0859-8 25776799

[bibr19-17470218241283028] Greve A. Cooper E. Tibon R. Henson R. N. (2019). Knowledge is power: Prior knowledge aids memory for both congruent and incongruent events, but in different ways. Journal of Experimental Psychology. General, 148(2), Article 2. 10.1037/xge0000498 PMC6390882 30394766

[bibr20-17470218241283028] Heinen R. Bierbrauer A. Wolf O. T. Axmacher N. (2024). Representational formats of human memory traces. Brain Structure and Function, 229, 513–529. 10.1007/s00429-023-02636-9 37022435 PMC10978732

[bibr21-17470218241283028] Höltje G. Lubahn B. Mecklinger A. (2019). The congruent, the incongruent, and the unexpected: Event-related potentials unveil the processes involved in schematic encoding. Neuropsychologia, 131, 285–293. 10.1016/j.neuropsychologia.2019.05.013 31112723

[bibr22-17470218241283028] Hovhannisyan M. Clarke A. Geib B. R. Cicchinelli R. Monge Z. Worth T. Szymanski A. Cabeza R. Davis S. W. (2021). The visual and semantic features that predict object memory: Concept property norms for 1,000 object images. Memory & Cognition, 49(4), Article 4. 10.3758/s13421-020-01130-5 PMC8081674 33469881

[bibr23-17470218241283028] Khosla A. Raju A. S. Torralba A. Oliva A. (2015). Understanding and predicting image memorability at a large scale. In 2015 IEEE International Conference on Computer Vision (ICCV) (pp. 2390–2398). Institute of Electrical and Electronics Engineers. 10.1109/ICCV.2015.275

[bibr24-17470218241283028] Krugliak A. Draschkow D. Võ M. L.-H. Clarke A. (2023). Semantic object processing is modulated by prior scene context. Language, Cognition and Neuroscience, 39(8), 962–971. 10.1080/23273798.2023.2279083 39404678 PMC7616603

[bibr25-17470218241283028] Lauer T. Cornelissen T. H. W. Draschkow D. Willenbockel V. Võ M. L.-H. (2018). The role of scene summary statistics in object recognition. Scientific Reports, 8(1), Article 1. 10.1038/s41598-018-32991-1 PMC6168578 30279431

[bibr26-17470218241283028] Moss H. E. Tyler L. K. Taylor K. I. (2007). Conceptual structure. In Gaskell G. (Ed.), Oxford handbook of psycholinguistics (pp. 217–234). University Press.

[bibr27-17470218241283028] Naspi L. Hoffman P. Devereux B. Thejll-Madsen T. Doumas L. A. A. Morcom A. (2021). Multiple dimensions of semantic and perceptual similarity contribute to mnemonic discrimination for pictures. Journal of Experimental Psychology: Learning, Memory, and Cognition, 47(12), Article 12. 10.1037/xlm0001032 34472918

[bibr28-17470218241283028] Oliva A. Torralba A. (2007). The role of context in object recognition. Trends in Cognitive Sciences, 11(12), Article 12. 10.1016/j.tics.2007.09.009 18024143

[bibr29-17470218241283028] Palmer tephen E . (1975). The effects of contextual scenes on the identification of objects. Memory & Cognition, 3(5), Article 5. 10.3758/BF03197524 24203874

[bibr30-17470218241283028] Quent J. A. Greve A. Henson R. N. (2022). Shape of U: The nonmonotonic relationship between object–location memory and expectedness. Psychological Science, 33(12), 2084–2097. 10.1177/09567976221109134 36221196

[bibr31-17470218241283028] Reed Hunt R . (2006). The concept of distinctiveness in memory research. In Hunt R. R. Worthen J. B. (Eds.), Distinctiveness and Memory (pp. 2–25). Oxford University Press. 10.1093/acprof:oso/9780195169669.003.0001

[bibr32-17470218241283028] Roediger H. L. Watson J. M. McDermott K. B. Gallo D. A. (2001). Factors that determine false recall: A multiple regression analysis. Psychonomic Bulletin & Review, 8(3), 385–407. 10.3758/BF03196177 11700893

[bibr33-17470218241283028] Souza C. Garrido M. V. Horchak O. V. Carmo J. C. (2022). Conceptual knowledge modulates memory recognition of common items: The selective role of item-typicality. Memory & Cognition, 50(1), 77–94. 10.3758/s13421-021-01213-x 34363197

[bibr34-17470218241283028] Standing L. Conezio J. Haber R. N. (1970). Perception and memory for pictures: Single-trial learning of 2500 visual stimuli. Psychonomic Science, 19(2), 73–74. 10.3758/BF03337426

[bibr35-17470218241283028] Staresina B. P. Gray J. C. Davachi L. (2009). Event congruency enhances episodic memory encoding through semantic elaboration and relational binding. Cerebral Cortex, 19(5), 1198–1207. 10.1093/cercor/bhn165 18820289 PMC2665161

[bibr36-17470218241283028] Taylor K. I. Devereux B. J. Acres K. Randall B. Tyler L. K. (2012). Contrasting effects of feature-based statistics on the categorisation and identification of visual objects. Cognition, 122(3), Article 3. 10.1016/j.cognition.2011.11.001 PMC3601414 22137770

[bibr37-17470218241283028] Taylor K. I. Devereux B. J. Tyler L. K. (2011). Conceptual structure: Towards an integrated neurocognitive account. Language and Cognitive Processes (Cognitive Neuroscience of Language), 26(9), Article 9. 10.1080/01690965.2011.568227 PMC3673226 23750064

[bibr38-17470218241283028] Tyler L. K. Chiu S. Zhuang J. Randall B. Devereux B. J. Wright P. Clarke A. Taylor K. I. (2013). Objects and categories: Feature statistics and object processing in the ventral stream. Journal of Cognitive Neuroscience, 25(10), Article 10. 10.1162/jocn_a_00419 PMC3767967 23662861

[bibr39-17470218241283028] van Kesteren M. T. R. Beul S. F. Takashima A. Henson R. N. Ruiter D. J. Fernández G . (2013). Differential roles for medial prefrontal and medial temporal cortices in schema-dependent encoding: From congruent to incongruent. Neuropsychologia, 51(12), Article 12. 10.1016/j.neuropsychologia.2013.05.027 23770537

[bibr40-17470218241283028] van Kesteren M. T. R. Ruiter D. J. Fernández G. Henson R. N . (2012). How schema and novelty augment memory formation. Trends in Neurosciences, 35(4), Article 4. 10.1016/j.tins.2012.02.001 22398180

[bibr41-17470218241283028] Võ M. L.-H. (2021). The meaning and structure of scenes. Vision Research, 181, 10–20. 10.1016/j.visres.2020.11.003 33429218

[bibr42-17470218241283028] Xiao J. Hays J. Ehinger K. A. Oliva A. Torralba A. (2010). SUN database: Large-scale scene recognition from abbey to zoo. In 2010 IEEE Computer Society Conference on Computer Vision and Pattern Recognition (pp. 3485–3492). Institute of Electrical and Electronics Engineers. 10.1109/CVPR.2010.5539970

